# Repurposing Drugs in Controlling Recurrent Platinum-Resistant Clear-Cell Ovarian Cancer

**DOI:** 10.1155/2023/2079654

**Published:** 2023-06-07

**Authors:** Woraporn Sinsuwan, Phawit Norchai

**Affiliations:** College of Integrative Medicine (CIM), Dhurakij Pundit University, Thailand

## Abstract

**Background:**

Recurrent platinum-resistant clear-cell ovarian cancer has a low overall survival duration of 7-8 months, making it a fatal disease. Currently, chemotherapy is the major kind of treatment, but it offers little advantage. Repurposed conventional drugs have recently been found to offer the ability to control cancer with few side effects and at a reasonable cost to healthcare organizations. *Case Presentation*. In this case report, we present the case of a 41-year-old Thai female patient diagnosed with recurrent platinum-resistant clear-cell ovarian cancer (PRCCC) in the year 2020. After undergoing chemotherapy for two courses and failing to respond to treatment, she began alternative medicine with repurposing drugs in November 2020. Simvastatin, metformin, niclosamide, mebendazole, itraconazole, loratadine, and chloroquine were also administered. Two months after therapy, a computerized tomography (CT) scan revealed a conflict between a decline in tumor marker levels (CA 125, CA 19-9) and an increase in the number of lymph nodes. However, after continuing all medications for 4 months, the CA 125 level decreased from 303.6 to 54 U/ml, and the CA 19-9 level decreased from 1210.3 to 386.10 U/ml. The patient's EQ-5D-5L score increased from 0.631 to 0.829 (abdominal pain and depression), indicating improved quality of life. Overall survival was 8.5 months, and progression-free survival was 2 months.

**Conclusion:**

The response to drug repurposing is demonstrated by a four-month-long improvement in symptoms. This work introduces a novel strategy for the management of recurrent platinum-resistant clear-cell ovarian cancer that needs further evaluation in large-scale studies.

## 1. Introduction

Advanced ovarian cancer is a very aggressive tumor and ranks fifth in cancer deaths among women. Treatment options in the recurrent setting are limited, especially in platinum-resistant patients who have a poor prognosis and a short survival time. These patients can enroll in clinical studies, try different chemotherapy regimens, or take advantage of the best programs for supportive care [1–3]. With a median overall survival (OS) of fewer than 12 months and a median progression-free survival (PFS) of 3 months, the response rate to the next course of chemotherapy is 15-20% [4].

It typically takes 10–17 years to create a new anticancer medicine from testing to licensure, and additional time is needed for commercial acceptance [5]. Advanced cancer patients only have a short window of time to wait for cutting-edge new anticancer medications.

The idea of repurposing medications entails using already approved medications for new uses. In oncology, it means using noncancer drugs to treat cancer. Thalidomide is an example of a medicine that has been repurposed; it was originally prescribed as a sedative to treat morning sickness in pregnant women. However, thalidomide was pulled off the market because we knew it had harmful negative effects on fetuses. Nevertheless, thalidomide was later approved for the treatment of multiple myeloma [6–8]. Drug development might take less time and expense with the use of molecular docking techniques. Molecular docking is a technique used to anticipate the orientation, contact, and affinity of the ligand-target complex. Noncancer medications are investigated using molecular docking techniques to locate target proteins in cancer pathways based on the current understanding of carcinogenesis and molecular pathways [7, 9].

The use of repurposed medications for ovarian cancer patients is not a recent development. Ritonavir, ivermectin, and COX-2 inhibitors are some of the older medications being studied by researchers to see if they have any anticancer benefits for ovarian cancer [10, 11]. The effectiveness of repurposing drugs like statins, metformin, or itraconazole alone or in combination with chemotherapy for advanced ovarian cancer has been the subject of numerous clinical investigations [12, 13].

In this case report, we offer a novel method for treating patients with recurrent platinum-resistant clear-cell ovarian cancer (PRCCC) by combining several repurposed nononcology medications.

## 2. Case Presentation

The patient, a 41-year-old woman, was found to have an early-stage clear-cell carcinoma of the right ovary, in addition to being an inactive chronic hepatitis B carrier. In April 2019, she started to have sporadic pelvic pain. Endometriosis was not a medical history. A solitary mass was detected by ultrasound in the right ovary at the hospital. Increased amounts of CA 125 and CA 19-9 levels were found in the blood sample. In October 2019, she underwent cytoreductive surgery and six cycles of adjuvant platinum-based chemotherapy. The result was a complete remission.

The patient developed recurrent disease eight months later, showing signs of raised blood tumor markers and a rise in the quantity of intra-abdominal lymph nodes. The hospital's doctor restarted platinum-based chemotherapy, but the patient experienced deep vein thrombosis in her left leg and paclitaxel hypersensitivity during the third cycle. After three cycles of chemotherapy, the advancement of the disease on a CT scan is allowed for the diagnosis of platinum drug resistance. The last dose was administered in September 2020. Due to side effects, the patient declined more chemotherapy and sought other treatments.

The patient came to our clinic in November 2020 with Eastern Cooperative Oncology Group (ECOG) performance status 1 [14]. She still complained of sporadic abdominal pain and depression. On a physical exam, her vital signs were stable. She was thin and mildly pale, with a mild degree of left leg swelling. Her current medications and supplements were rivaroxaban (15 mg/day), clonazepam (2 mg/day), trazodone (50 mg/day), nortriptyline (10 mg/day), beta-glucan, and apricot. Her prior blood tests revealed that her kidney and liver functions were normal. The hospital did not perform any cancer molecular testing or other specialized marker tests due to the resource-limited setting and the high cost of drugs, which patients cannot afford.

The treatment program consisted of 5 weeks at our clinic in Bangkok, followed by continued use of only repurposing drugs and dietary supplements at home, a phone check-in, and a referral for blood tests every two weeks.

The patient gave their consent after being fully briefed at the clinic. Every two weeks, she underwent a physical examination and blood tests. The current quality of life was assessed using the EQ-5D-5L Thai version questionnaire, and it was then repeated after 4 months [15].

Following a review of potential drug interactions between repurposing drugs and the patient's existing prescriptions, we began giving the patient simvastatin (20 mg/day) in the second week after first prescribing metformin (500 mg/day), mebendazole (500 mg/week), loratadine (10 mg/day), and niclosamide (2 gm/day) in the first week. In the fourth week, itraconazole (100 mg/day) and chloroquine (250 mg every other day) were both administered. In addition, she received homeopathy, oxygen treatment, lymphatic therapy, high-dose intravenous vitamin C, and mental support as required at the clinic. At the commencement of the treatment, high-potency supplements containing vitamins, minerals, melatonin, armor thyroid, probiotics, and phytochemicals were started. These included novasol curcumin (3 gm/day), quercetin (2000 mg/day), bromelain (6 gm/day), and milk thistle (750 mg/day).

As the treatment regimen continued, her abdominal pain, swelling in her left leg, and depression showed improvement, and she was able to stop antidepressant drugs. Serologically, her CA 125 level began to decrease after 2 weeks of the program and continued to decline. In the eightieth week, after she returned home, her CA 125 level decreased from 303.6 to 75 U/ml (Table 1). We confirmed the response with a CT scan of the abdomen at a local hospital. A CT scan, which was compared between September 27, 2020 and January 6, 2021, showed an increase in both size and number of matted lymph nodes predominating at the upper abdomen and partial-recanalized chronic thrombosis without organ distant metastasis. Her symptoms were improved, so we suggested she continue taking oral supplements and medications.

The EQ-5D-5L score increased from 0.631 to 0.829 after 4 months of therapy, which decreased pain and depression (Table 2). Along with the decline in tumor markers, the levels of CA 19-9 and CA 125 fell to 54 U/ml and 386.10 U/ml, respectively.

She experienced sporadic abdominal pain again at the end of the fifth month, and a blood test revealed elevated levels of CA 125 and CA 19-9 at 181.9 U/ml and 925.2 U/ml, respectively. The patient made the decision to discontinue all oral medications temporarily after we discussed drug modifications with her. She passed away in the middle of July 2021.

## 3. Discussion

This case report shows that patients with recurrent platinum-resistant clear-cell ovarian cancer may be able to manage their symptoms and improve their quality of life with a combination of repurposing drugs. The patient was able to perform daily tasks and go back to work normally without experiencing any severe toxicities.

PRCCC, which stands for platinum-resistant clear-cell ovarian cancer, is characterized by its limited response to chemotherapy and poor prognosis with a short survival time [16]. This type of cancer often affects younger patients. Hemman and Rattanaburi conducted a study in the Thai population and found that patients with recurrent PRCCC who received an additional line of chemotherapy had an estimated survival time of 7.8 months [17]. The results of our patient align with these findings, as she had a progression-free survival of 2 months and an overall survival of 8.5 months.

After applying the Gynecologic Cancer InterGroup (GCIG) CA 125 criteria to our patient to evaluate response in the relapse setting, we observed that the disease response began on December 23, 2020. A 50% reduction in the baseline CA 125 level (303.6 U/ml to 75 U/ml) was evident, and a confirmatory test conducted on January 28, 2021 showed a level (81 U/ml) that was within 10% of the previous test. We had to combine the findings of the CT scan according to Response Evaluation Criteria in Solid Tumours (RECIST) [18] and the blood CA 125 level together in order to meet the criteria for the GCIG evaluation for “best overall response” [19, 20], and the outcome of combining both criteria was a progression of the disease at two months.

Since controlling the disease and its symptoms, including toxicities from treatment, and improving quality of life are the ultimate goals of managing recurrent metastatic cancer [21, 22], we decided to continue medications when our patient reported that she was in better health and had no toxicity from treatment. The level of CA 125 decreased from 81 U/ml to 54 U/ml after using repurposed medications for 4 months, and the EQ-5D-5L score revealed improvements in pain and depression.

The conflicting results between the reduction of CA 125 levels and the progression of lymph nodes on a CT scan could be explained by two possibilities. Firstly, repurposing drugs may work in a similar manner to biological therapy [23] by blocking multiple hallmarks of cancer [24], such as controlling tumor metabolism, inhibiting tumor proliferation [5, 10, 13], and decreasing inflammation, which can enhance the body's immunity [25]. This kind of therapy helps to slow tumor growth and reduce tumor activity rather than decrease tumor size; hence, repurposing drugs might not show tumor response by RECIST criteria. Secondly, the heterogeneity of tumor mass [26] could contribute to different responses, especially considering the exposure to multiple lines of chemotherapy or radiation, which can lead to the emergence of new mutations. While some cancer cells may respond to the treatment, others may not, posing a challenge for physicians to overcome. This heterogeneity within the tumor can account for the divergent outcomes observed in the patient's case.

After 3 months of treatment, the CA 19-9 level was still higher than the beginning level. One study showed that CA 19-9 levels were higher in the metastatic setting compared to the occult primary setting in ovarian cancer [27]. In our patient, it appeared that the repurposing drugs were effective in lowering CA 19-9 levels, but they were insufficient to stop the mechanism of metastasis because the final CT scan revealed numerous more upper abdominal lymph nodes.

After taking repurposed drugs for five months (through March 31, 2021), the patient experienced sporadic abdominal pain, and the edema in her left leg returned. Both the CA 125 and CA 19-9 levels had risen from 54 U/ml to 181.9 U/ml and 386.10 U/ml to 925.2 U/ml, respectively. The patient passed away in the middle of July 2021 after asking for all treatments to stop.

Recently, Goenka et al. summarized clinical studies on repurposing drugs. The old generic medications that had been looked into included metformin, statins, proton pump inhibitors, itraconazole, and beta-blockers. The study's approach involved evaluating each medication either alone or in combination with chemotherapy. Most of the studies were in first-line settings [13]. Our study is the first case report testing the efficacy of combined multiple repurposing drugs in a recurrent setting of platinum-resistant clear-cell ovarian cancer. According to the hallmarks of cancer, we hypothesized that using multiple drugs to block most cancer pathways might result in the desired outcome.

This case report selected the study drugs from literature reviews that showed potential efficacy in controlling ovarian cancer, for example, metformin, statin, itraconazole, and chloroquine [12, 13]. We additionally included loratadine [28], mebendazole [5, 29, 30], and niclosamide [5, 31] to inhibit the growth, invasion, and metastasis of cancer cells, as well as their ability to repair DNA damage. According to a clear-cell subtype molecular study, ARID1A and PIK3CA mutations occur in 50% of cases. Metformin, statin, and itraconazole have the potential to block these PIK3CA mutation pathways [5, 32–34].

Developing countries, including Thailand, lack access to all drugs in the guidelines, especially expensive targeted therapy, and immunotherapy-related drugs. Therefore, researching the anticancer effect of repurposing drugs might result in a new alternative therapy for recurrent platinum-resistant clear-cell ovarian cancer patients.

The study was limited by telemedicine and the COVID-19 pandemic year; as a result, we were unable to perform a physical examination or assess the patient's adherence to medication. When the patient's illness progressed, we were also unable to ask them to visit the clinic for an examination because of the COVID-19 outbreak in Thailand.

## 4. Conclusion

There are currently few and ineffective treatments for recurrent platinum-resistant clear-cell ovarian cancer. Combining repurposing drugs showed tumor response by lowering CA125 levels and improving quality of life. Repurposing drugs also exhibited low toxicity profiles and was a well-tolerated treatment in addition to the potential effects. A well-designed prospective study is needed to confirm the efficacy of the combined repurposing of medications, which seems to be a novel approach.

## Figures and Tables

**Table 1:** Level of blood CA 125 and CA 19-9.

Tumor markers	1 November 2020	7 November 2020	23 November 2020	7 December 2020	23 December 2020	28 January 2021	23 February 2021	31 March 2021
CA 125 (U/ml)	303.6	Started program	230	192	75	81	54	181.9
CA 19-9 (U/ml)	1210.3		3470	2702.71	1522.60	1559.40	386.10	925.2

**Table 2:** Evaluate quality of life by EQ-5D-5L Thai version questionnaire.

Health dimension	Before treatment	After 4 months of treatment
Mobility	2	2
Self-care	1	1
Usual activities	2	2
Pain/discomfort	3	2
Anxiety/depression	4	2
Utility score of the survey respondent	0.631	0.829

## Data Availability

The data that support the findings of this report are included in this article.

## References

[1] Doubeni C. A., Doubeni A. R. B., Myers A. E. (2016). Diagnosis and management of ovarian cancer. *American Family Physician*.

[2] Pignata S., Cecere S. C., du Bois A., Harter P., Heitz F. (2017). Treatment of recurrent ovarian cancer. *Annals of Oncology*.

[3] National Comprehensive Cancer network (2022). Ovarian cancer including fallopian tube cancer and primary peritoneal cancer (version 5.2022). https://www.nccn.org/professionals/physician_gls/pdf/ovarian.pdf.

[4] Davis A., Tinker A. V., Friedlander M. (2014). “Platinum resistant” ovarian cancer: what is it, who to treat and how to measure benefit?. *Gynecologic Oncology*.

[5] Zhang Z., Zhou L., Xie N. (2020). Overcoming cancer therapeutic bottleneck by drug repurposing. *Signal Transduction and Targeted Therapy*.

[6] Verbaanderd C., Meheus L., Huys I., Pantziarka P. (2017). Repurposing drugs in oncology: next steps. *Trends in Cancer*.

[7] Pushpakom S., Iorio F., Eyers P. A. (2019). Drug repurposing: progress, challenges and recommendations. *Nature Reviews Drug Discovery*.

[8] Shah R. R., Stonier P. D. (2019). Repurposing old drugs in oncology: opportunities with clinical and regulatory challenges ahead. *Journal of Clinical Pharmacy and Therapeutics*.

[9] Turanli B., Grøtli M., Boren J. (2018). Drug repositioning for effective prostate cancer treatment. *Frontiers in Physiology*.

[10] Banno K., Iida M., Yanokura M. (2015). Drug repositioning for gynecologic tumors: a new therapeutic strategy for cancer. *Scientific World Journal*.

[11] Nunes M., Abreu M. H., Bartosch C., Ricardo S. (2020). Recycling the purpose of old drugs to treat ovarian cancer. *International Journal of Molecular Sciences*.

[12] Difeo A. (2019). Repurposed drugs trials for ovarian cancer. *The Cancer Journal*.

[13] Goenka L., Dubashi B., Selvarajan S., Ganesan P. (2022). Use of “repurposed” drugs in the treatment of epithelial ovarian cancer. *American Journal of Clinical Oncology*.

[14] Azam F., Latif M. F., Farooq A. (2019). Performance status assessment by using ECOG (Eastern Cooperative Oncology Group) score for cancer patients by oncology healthcare professionals. *Case Reports in Oncology*.

[15] Pattanaphesaj J., Thavorncharoensap M., Tongsiri S., Ingsrisawang L., Teerawattananon Y. (2018). The EQ-5D-5L valuation study in Thailand. *Expert Review of Pharmacoeconomics & Outcomes Research*.

[16] Itamochi H., Kigawa J., Terakawa N. (2008). Mechanisms of chemoresistance and poor prognosis in ovarian clear cell carcinoma. *Cancer Science*.

[17] Hemman W., Rattanaburi A. (2022). Incidence and predictive factors for recurrent clear cell ovarian carcinoma: results from a single center in Thailand. *Obstetrics & Gynecology Science*.

[18] Eisenhauer E. A., Therasse P., Bogaerts J. (2009). New response evaluation criteria in solid tumours: revised RECIST guideline (version 1.1). *European Journal of Cancer*.

[19] The Gynecological Cancer InterGroup (2005). CA 125 definitions agreed by GCIG November 2005. https://gcigtrials.org/system/files/CA%20125%20Definitions%20Agreed%20to%20by%20GCIG%20%20November%202005.pdf.

[20] Stuart G. C. E., Kitchener H., Bacon M. (2011). 2010 Gynecologic Cancer InterGroup (GCIG) consensus statement on clinical trials in ovarian cancer: report from the fourth ovarian cancer consensus conference. *International Journal of Gynecological Cancer*.

[21] Lipscomb J., Gotay C. C., Snyder C. F. (2007). Patient-reported outcomes in cancer: a review of recent research and policy initiatives. *CA: a Cancer Journal for Clinicians*.

[22] Gotay C. C., Kawamoto C. T., Bottomley A., Efficace F. (2008). The prognostic significance of patient-reported outcomes in cancer clinical trials. *Journal of Clinical Oncology*.

[23] Bookman M. A. (1998). Biological therapy of ovarian cancer: current directions. *Seminars in Oncology*.

[24] Hanahan D. (2022). Hallmarks of cancer: new dimensions. *Cancer Discovery*.

[25] Ben-Baruch A. (2006). Inflammation-associated immune suppression in cancer: the roles played by cytokines, chemokines and additional mediators. *Seminars in Cancer Biology*.

[26] Janku F. (2014). Tumor heterogeneity in the clinic: is it a real problem?. *Therapeutic Advances in Medical Oncology*.

[27] Sagi-Dain L., Lavie O., Auslander R., Sagi S. (2015). CA 19-9 in evaluation of adnexal mass: retrospective cohort analysis and review of the literature. *The International Journal of Biological Markers*.

[28] Fritz I., Wagner P., Olsson H. (2021). Improved survival in several cancers with use of H_1_-antihistamines desloratadine and loratadine. *Translational Oncology*.

[29] Huang L., Zhao L., Zhang J. (2021). Antiparasitic mebendazole (MBZ) effectively overcomes cisplatin resistance in human ovarian cancer cells by inhibiting multiple cancer-associated signaling pathways. *Aging*.

[30] Mansoori S., Fryknäs M., Alvfors C., Loskog A., Larsson R., Nygren P. (2021). A phase 2a clinical study on the safety and efficacy of individualized dosed mebendazole in patients with advanced gastrointestinal cancer. *Scientific Reports*.

[31] Shangguan F., Liu Y., Ma L. (2020). Niclosamide inhibits ovarian carcinoma growth by interrupting cellular bioenergetics. *Journal of Cancer*.

[32] Wang T., Seah S., Loh X. (2016). Simvastatin-induced breast cancer cell death and deactivation of PI3K/Akt and MAPK/ERK signalling are reversed by metabolic products of the mevalonate pathway. *Oncotarget*.

[33] Tsubamoto H., Inoue K., Sakata K. (2017). Itraconazole inhibits AKT/mTOR signaling and proliferation in endometrial cancer cells. *Anticancer Research*.

[34] Iida Y., Okamoto A., Hollis R. L., Gourley C., Herrington C. S. (2021). Clear cell carcinoma of the ovary: a clinical and molecular perspective. *International Journal of Gynecological Cancer*.

